# Driving Pest Insect Populations: Agricultural Chemicals Lead to an Adaptive Syndrome in *Nilaparvata Lugens* Stål (Hemiptera: Delphacidae)

**DOI:** 10.1038/srep37430

**Published:** 2016-11-23

**Authors:** Lin-Lin You, You Wu, Bing Xu, Jun Ding, Lin-Quan Ge, Guo-Qin Yang, Qi-Sheng Song, David Stanley, Jin-Cai Wu

**Affiliations:** 1 School of Plant Protection, Yangzhou University, Yangzhou 225009, China; 2 Division of Plant Sciences, University of Missouri, Agriculture Building, Columbia, Mo 65211, USA.; 3 USDA/Agricultural Research Service, Biological Control of Insects Research Laboratory, Columbia, Missouri, MO, 65211, USA.

## Abstract

The brown planthopper (BPH) is a devastating pest of rice throughout Asia. In this paper we document the BPH biogeographic range expansion in China over the 20-year period, 1992 to 2012. We posed the hypothesis that the range expansion is due to a syndrome of adaptations to the continuous presence of agricultural chemicals (insecticides and a fungicide) over the last 40 years. With respect to biogeography, BPH ranges have expanded by 13% from 1992 to 1997 and by another 3% from 1997 to 2012. In our view, such expansions may follow primarily from the enhancing effects of JGM, among other agricultural chemicals, and from global warming. JGM treatments led to increased thermotolerance, recorded as decreased mortality under heat stress at 40 ± 1 °C (down from 80% to 55%) and increased fecundity (by 49%) at 34 °C. At the molecular level, JGM treatments led to increased abundances of mRNA encoding Acetyl Co-A carboxylase (Acc) (up 25%) and Hsp70 (up 32%) in experimental BPH. RNAi silencing of *Hsp70* and *Acc* eliminated the JGM effects on fecundity and silencing *Hsp70* reduced JGM-induced thermotolerance. Integrated with global climate change scenarios, such syndromes in pest insect species have potential for regional- and global-scale agricultural disasters.

Approximately 11% of the 13.4 billion hectares (ha) of the earth’s land is devoted to agriculture, which is projected to decline slightly over the next half century^1^. Such large scale human activities impact the ecology and biogeography of many organisms, including insects, at the global level. Due to their relatively rapid life cycles and intense selection pressure, insect pests have enormous capacity to evolve adaptive mechanisms to thrive under the combined pressures of continued global warming and contemporary agricultural practices. Sophisticated analyses of global surface temperatures leave no doubt that in the last 100 years, the Earth’s temperature has increased by 0.7 °C, about 10 times faster than the rate of global temperature increase since the last ice-age^2^,^3^. The relatively rapid warming will lead to serious consequences in forest^4^ and agricultural ecosystems^5^. For one, species range-shifts occur rapidly, increasing in elevation by 11.0 m/decade and in latitudes by almost 17 km/decade^6^. Guo *et al*.^7^ predicted that whitefly (*Bemisia tabaci*, MEAM1) populations can withstand stress associated with increased temperatures and will expand northward under global warming. Such climate change can influence developmental rates, which can create phenological mismatches that separate pests from their natural enemies in time, with concomitant loss of biocontrol services^8^. Aside from the issues associated with global climate patterns, insects are well adapted to human agriculture. The most common adaptive mechanism may be straightforward insecticide resistance. The beet armyworm, for example, has evolved over 3,000-fold resistance to the pyrethroid insecticide lambda cyhalothrin^9^. In extreme cases, insecticide resistance can lead to local disasters. For a single example, high resistance to methyl parathion in tobacco budworms led to the collapse of cotton production in southern Tamaulipas, Mexico^10^, which was later revitalized with the advent of Bollgard cotton. The concern, of course, is that insect adaptations to other agricultural practices have potential for larger-scale disasters, which can threaten food and nutrition security at the regional, national and international levels. Several mechanisms are pertinent. Pest population resurgence following insecticide applications was recorded soon after the beginning of the chemical insecticide era^11^. Sub-lethal insecticide exposures can lead to increased reproduction and pest population sizes and to behavioral changes^11^. The possibility that exposure to agricultural chemicals can led to increased reproduction and thermotolerance in some insect pests, under the current trend of global warming, is a recent and particular worry.

The brown planthopper (BPH), *Nilaparvata lugens*, is a geographically cosmopolitan insect and a serious rice pest in Asia, responsible for direct rice plant damage and for transmitting rice plant pathogenic viruses. It is a serious threat to sustainable rice production throughout its geographical range^12^,^13^. BPH outbreaks are associated with temperature, a key parameter for forecasting population outbreaks^14^. Taken with global warming and with the understanding that some insecticides lead to enhanced thermotolerance and reproduction in BPH^15^, we are investigating the molecular adaptations to some agricultural chemicals that may lead to catastrophic losses in Asian rice production. Two insecticides, deltamethrin and trizaophos (tzp), stimulate increased BPH reproduction, in part by increasing expression of a gene encoding vitellogenin (Vg)^16^. At the organismal level, tzp exposure leads to increased ovarian protein contents and fecundity, recorded as egg laying^17^. Tzp treatments lead to substantial increases in thermotolerance, registered as reduced mortality at 40 °C and increased female lethal mean time (LT_50_) values. To investigate this at the molecular level, we designed dsRNA constructs to silence two BPH genes, one encoding a heat-shock 70 (Hsp70), a molecular chaperone, and one encoding arginine kinase (Argk) that effectively buffers ATP levels under some stressors. Individually silencing these two genes leads to elimination of tzp-induced thermotolerance in BPHs. Consistent with these results, tzp treatments led to increased expression of *Hsp70* and *Argk*. It can be inferred that tzp induces thermotolerance by increasing expression of genes acting in cell protection mechanisms^15^.

Aside from insecticides, another agriculture chemical, jinggangmycin (JGM), is a widely applied fungicide, used during every growing season over several decades to control rice sheath blight *Rhizoctonia solani*in China^18^. The fungicide is commonly applied two or three times during rice growth (mainly at the late tillering and booting stages) with leaf spray at the commercial rate of 125–175 g.a.i.ha^−1^. In effect, JGM has been a stable component of planthopper habitats for a very long time. Planthoppers make up an ecological guild composed of several species operating very similar niches. Two planthopper species, BPHs and white-backed planthoppers (WBPH), *Sogatella furcifera*, have evolved separate adaptations to JGM. The fungicide stimulates fecundity in BPH, but not WBPH^19^. The influence of JGM on BPH fecundity appears to act through several enzymes. Acetyl-CoA carboxylase (Acc)^20^ and fatty acid synthase (FAS)^21^ act in fatty acid synthesis and separately silencing genes encoding these enzymes eliminates the JGM-enhanced BPH fecundity. This work helps to understand the adaptive mechanisms planthoppers have evolved that lead to chemically-induced enhancements inBPH fecundity. The differences between BPH and WBPH demonstrate the operation of molecular niche separation, in which individual species within the planthopper guild have evolved different adaptations to agricultural chemicals at the molecular level^20^.

In this paper we document the BPH biogeographic range expansion in China over the 20- year period, 1992 to 2012. The range expansion could be due to global warming, however, we considered that unlikely due to the relatively slow warming rate compared to the fairly rapid range expansion. We posed the hypothesis that the expansion is due to a syndrome of adaptations to the continuous presence of agricultural chemicals (insecticides and a fungicide) over the last 40 years. JGM enhances fecundity and here we report that JGM also enhances BPH thermotolerance. The combined influences of enhanced fecundity and thermotolerance could lead to increased population sizes and biogeographic ranges. Here, we confirm our finding that JGM enhances fecundity, and report that the fungicide also enhances thermotolerance. The JGM influence can be seen at the organismal level in terms of tolerating heat stress and at themolecular level by separating the influences of two genes, *HSP70* and *Acc*, on thermotolerance and fecundity.

## Results

### BPH biogeography 1992–2012

**Figure 1:**
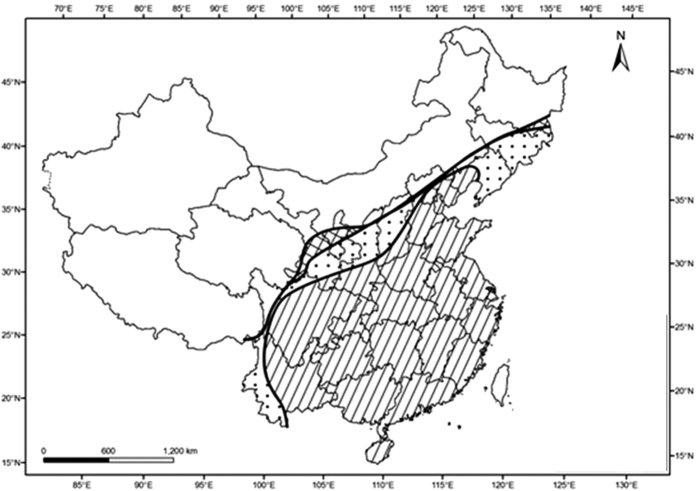
BPH biogeography in China mainland, years 1991, 1997, and 2012. Dots represent BPH range in 1997, grids represent BPH range in 2012. Historical BPH data were obtained from the Pest Forecasting Division of the National Agro-Tech Extension and Service Centre of China (NATESC), Ministry of Agriculture, China. The map is from the website http://www.diva-gis.org/Data. The image analysis was conducted in R using the Portable Network Graphics (png) and raster packages (R Code Team, 2015, http://www.R-project.org/). The software used to create the map is The Arclinfo GIS software (ESRI Inc., Redlands, California, USA) and Adobe Photoshop (Adobe System Software Ireland Ltd., Dublin, Ireland). QGIS Version 2.14 is downloaded from the website: http://www.qgis.org/en/site/forusers/download.html.

BPH distribution ranges expanded by 13% over the period 1991 to 1997 and by another 3% from 1997 to 2012 (Fig. 1). The rate of BPH expansion decreased in the second period, as the insects approached the western and northern ranges of Chinese rice planting area.

### JGM Enhances reproduction

**Figure 2:**
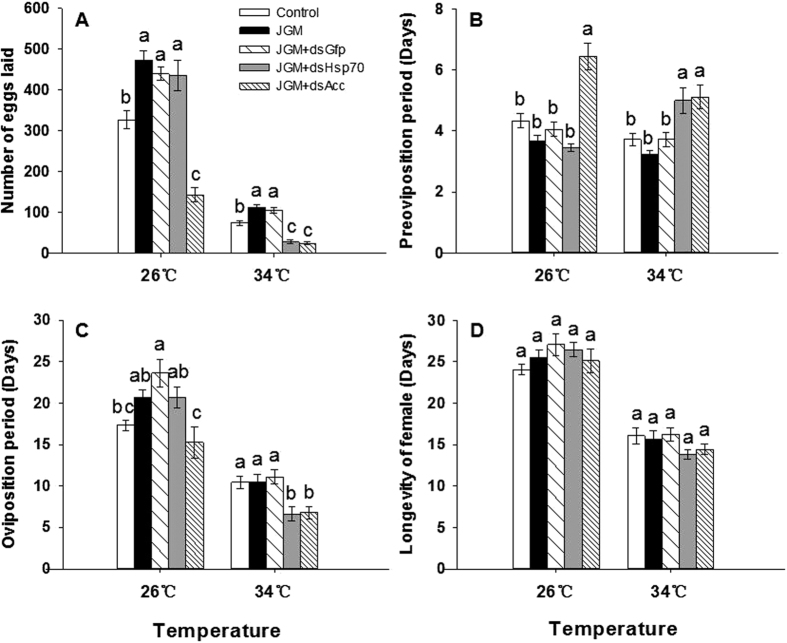
Influence of dsHsp70 and dsAcc treatments on BPH reproduction at 26 °C and 34 °C. Panel A: the number of eggs laid. Panel B: preoviposition period. Panel C: oviposition period. (**D**) female longevity. Bars annotated with the same letters within a temperature are not significantly different (P < 0.05). Each histogram bar represents mean ± S.E. Controls were untreated and JGM indicates the jinggangmycin-treated group. JGM + dsGfp, JGM + dsHsp70, and JGM + dsAcc represent a control dsRNA and*Hsp70* and *Acc* gene silencing treatments. n = three independent biological replicates, 18 adult females per biological replicate.

Compared to controls, JGM treatments led to increased fecundity, registered as numbers of eggs laid. At 26 °C, egg laying increased by 44.5%, from 328 to 474 eggs. Fecundity also increased by 49.3% in females held at 34 °C, albeit with a much reduced range from 75 to 112 eggs (Fig. 2A) (*P*-values listed in Table 1).

### Influence of suppressing Acc and Hsp70 expression on reproduction parameters

Compared to JGM-treated BPH, females treated with JGM + dsAcc laid far fewer eggs, down by 69.8% (Fig. 2A), from 474 to 143, at 26 °C, and down by 77.6%, from 112 to 25 eggs, at 34 °C. JGM + dsHsp70 treatments did not influence fecundity at 26 °C. At 34 °C, the JGM + dsHsp70 treatment led to reduced fecundity, down by 74.1%, from 112 to 29 eggs (Fig. 2A), compared to the results of JGM treatments. *Hsp70* and *Acc* silencing led to increased pre-oviposition periods (Fig. 2B; Table 1). JGM + dsAcc treatments led to increased pre-oviposition periods, up by 75.8% at 26 °C, compared to the results of JGM treatments. At 34 °C, JGM + dsHsp70 (↑55.2%) and JGM + dsAcc (↑58.6%) led to similar increases in pre-oviposition periods. In line with the influence of suppressing gene expression on pre-oviposition periods, JGM + dsAcc and JGM + dsHsp70 treatments led to abbreviated oviposition periods (Fig. 2C; Table 2). At 26 °C, JGM + dsAcc, but not dsHsp70, treatments led to decreased oviposition periods, down by 26.3%, compared to the results of JGM treatments. At 34 °C, JGM + dsHsp70 and JGM + dsAcc treatments led to decreased oviposition periods, down by about 35–37.0%, compared to the results of JGM treatments. Neither dsRNA treatment influenced female longevity (Fig. 2D; Table 1).

### JGM treatments enhance thermotolerance

**Figure 3:**
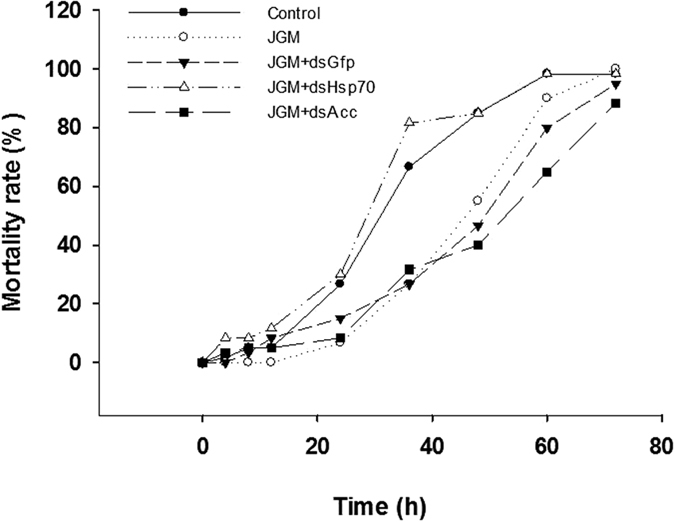
JGM treatments led to Hsp70-mediated increased thermotolerance, reported as proportional mortality. Comparing thecontrol and JGM-treated groups shows the JGM treatment increased survivorship. JGM + dsGfp, JGM + dsHsp70, and JGM + dsAcc represent a control dsRNA and *Hsp70* and *Acc* gene silencing treatments. n = 3 independent biological replicates, 60 adult females per biological replicate.

**Figure 4:**
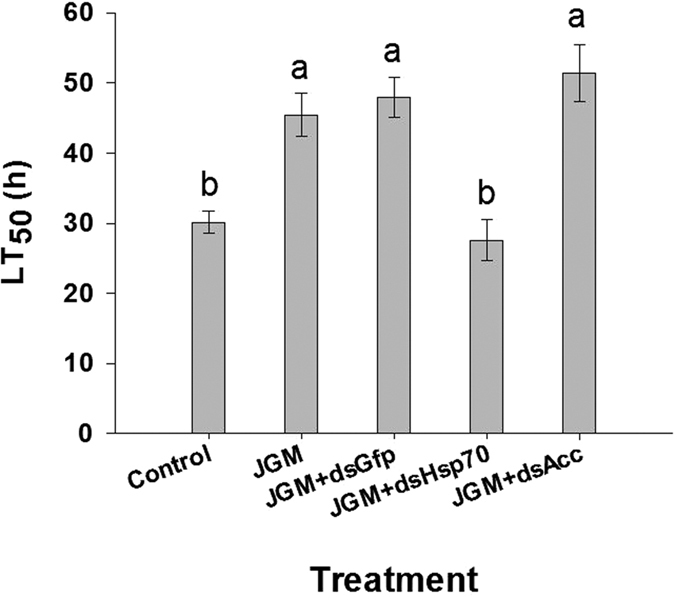
JGM treatments led to increased female lethal mean time (LT_50_) at 40 °C. Histogram bars indicate LT_50_ values in the indicated treatment groups. Bars annotated with the same letter are not significantly different (P < 0.05). Each histogram bar represents mean ± S.E. Comparing the control and JGM-treated groups shows the JGM treatment increased LT50 values. JGM + dsGfp, JGM + dsHsp70, and JGM + dsAcc represent a control dsRNA and *Hsp70* and *Acc* gene silencing treatments. n = 3 independent biological replicates, 60 adult females per biological replicate.

Mortality rates of JGM-treated adults following 48 h of temperature stress at 40 °C declined from about 80% in controls to approximately 55% in BPHs treated with JGM, JGM + dsGFP or JGM + dsAcc (Fig. 3). Suppressing *Hsp70* expression led to increased mortality, up about 42% to circa 80% after 48 h of temperature stress (Fig. 3), eliminating JGM-enhanced thermotolerance. Using LT_50_ values as a direct measure of thermotolerance at 40 °C, LT_50_ for untreated controls and for JGM + dsHsp70-treated experiments were similar at about 30 h while JGM, JGM + dsGFP and JGM + dsAcc treatments led to similar results, with LT_50_ values of about 45 h–50 h (Fig. 4; Table 1).

### JGM influences gene expression

**Figure 5:**
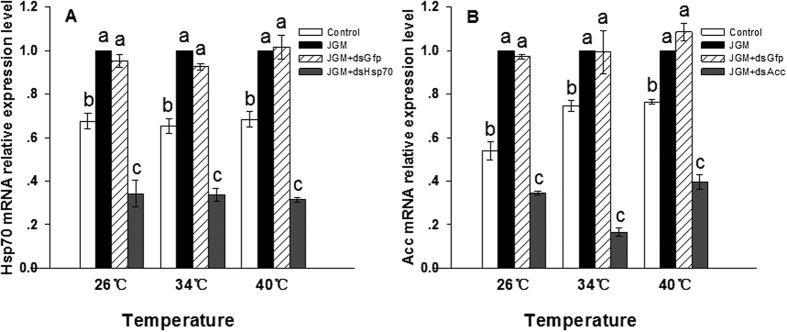
Hsp70 and Acc mRNA abundances at the indicated temperature in adult females emerged from 3^rd^ instar nymphs treated as shown on the panels. Panel A: Hsp70 transcript levels. Panel B: Acc transcript levels. Each histogram bar represents mean ± S.E., n = 3 independent biological replicates. Bars annotated with the same letters within each temperature are not significantly different (P < 0.05). The mRNA abundances were normalized relative to the β-actin transcript. Comparing the control and JGM-treated groups shows the JGM treatments led to increased accumulation of mRNA encoding Hsp70 and Acc. JGM + dsGfp represents a control dsRNA. Panel A shows the JGM + dsHsp70 treatment led to reduced Hsp70 mRNA accumulation. Panel B shown the JGM + dsAcc treatment led to reduced Acc mRNA accumulation. n = 3 independent biological replicates, five females per biological replicate.

JGM treatments led to increased expression of *Hsp70* (↑about 32%) and *Acc* (↑about 24%), compared to controls in experiments conducted at 40 °C (Fig. 5A,B). Suppressing expression of these two genes led to quantitative reductions in expression, *Hsp70* down by 69% and *Acc* down by about 61%, compared to the results of JGM treatments (Fig. 5A,B; Table 1). Similar patterns of JGM-stimulated and dsRNA-reduced gene expression were obtained from experiments conducted at 26 °C and 34 °C (Fig. 5).

## Discussion

In this work we document the expansion of BPH biogeography over a 20-year period and test our hypothesis that the expansion is due to a syndrome of adaptations to the continuous presence of agricultural chemicals (insecticides and a fungicide) over the last several decades. Several points are germane. First, JGM treatments led to decreased mortality rates and increased LT_50_ values in temperature-challenged BPHs. Second, dsRNA treatments demonstrate that the enhancement of JGM-induced fecundity operates through both *HSP70* and *Acc*, but the enhancement of JGM-induced thermotolerance operates through *Hsp70*, not *Acc*. Finally, JGM treatments, coupled with high temperature stress led to increased expression of *Hsp70* and *Acc*, which was abolished by the gene-suppression treatments. Taken together, these points form an argument for a syndrome of pest adaptations to presence of agricultural chemicals.

Induced thermotolerance has been reported in many organisms, including prokaryotes, plants, aquatic and marine vertebrates and invertebrates, as well as for insects. For example, larval density positively influences heat stress survival in *D. melanogaster* adults^22^. A correlation between *Hsp70* expression and thermotolerance in three developmental stages (late embryos, late larvae and adults), supported the inferrence that Hsp70 acts in induced thermotolerance in *C. capitata*^23^. Increasing the magnitude and duration of pretreatment induced higher Hsp70 concentrations and improved tolerance of severe stress, also in *Drosophila*^24^. After Hsp70 concentrations exceed a critical point, they found larval survival and cell growth declined. Based on the timing of mortality, the authors suggested that larvae die more rapidly from over-expression of Hsp70 than from direct heat damage. The authors concluded that precise regulation of Hsp concentrations is important for surviving potentially lethal temperatures^24^. Looking below the organismal level, Klose *et al*.^25^ showed that tolerating elevated temperatures in presynaptic nerve terminals in *Drosophila* is related to stability of the energy-requiring mechanisms that regulate intracellular ion concentrations rather than the usual chaperone proteins. Hence, our finding that exposure to JGM leads to enhanced thermotolerance is consistent with contemporary wisdom. Our demonstration that JGM acts via increasing *Hsp70* expression unveils a molecular mechanism of the JGM effects. We note that tzp treatments also led to increased thermotolerance; it is not surprising to see that both tzp and JGM operate via the same gene, *Hsp70*^15^.

The adaptive syndrome we propose is multi-faceted, including highly increased insecticide resistance^26^,^27^, increased flight capacity^28^, increased reproduction and population sizes and, as shown here, enhanced thermolerance^15^. The increased thermotolerance is noteworthy in light of the biology of BPH, which undergoes weather-dependent, windborne migrations. Although BPH is not a cold tolerant insect species, their relationships with agricultural chemicals may have already led to expanded BPH biogeography, which increased to the near the edges of rice cultivation in China over a 20-year span, as just reported. We note that BPH has substantial migratory capability, from overwintering areas in Vietnam to as many as 14 Chinese provinces and via overseas routes, to Taiwan, Korea and Japan^29^. In a given year, the BPH migration boundary is related to time-specific airstreams. Here we stress the idea that the growth of migrating populations mainly depends on the hypothesized adaptive syndrome. It follows that the pesticide-induced adaptive syndrome contributes to the geographical expansion of insect pests. Increased thermotolerance, as a component of the adaptive syndrome, may allow earlier seasonal migrations to more northern ranges via suitable prevailing winds because the adaptive syndrome could enhance BPH stress tolerance during and after migration. In addition, some agricultural practices, such as increased use of BPH-susceptible rice varieties and overlapping wheat, barley and rice culture, may facilitate increasing BPH ranges in other Asian regions. The BPH adaptive syndrome may become quite important because it was recently shown that BPH outbreaks in the Yangtze River Delta were due to very high reproductive rates, rather than migration^30^.

The idea that agricultural chemicals are responsible for increasing the biogeographic range of a pest species may seem at odds with the received wisdom that global warming has already led to expansions of many plant and animal species^6^. In our view, adaptation to constant exposure to agricultural chemicals and the influence of warming environments are two of many forces responsible for biogeography of organisms. We emphasize the significance of adapting to agricultural chemicals because pest-favoring adaptive syndromes, coupled with global warming, may become very potent selective forces.

JGM treatments also led to increased fecundity, as we recently reported^20^,^21^. Taken with the data reported here, these papers provide new insights into the influence of agricultural chemicals on BPH biology. First, we note that the dsAcc treatments described here influenced fecundity, but not thermotolerance in experimental BPH. JGM exposure also enhances fecundity via its influence on expression of *Acc* and *Fas*. Both of these genes mediate the biosynthesis of fatty acids, which are central actors in reproductive biology, providing biochemical signaling, energy and chemical structures necessary for ovarian and follicle maturation. It is reasonable to suppose that JGM influences fecundity through these as well as other genes awaiting discovery. dsAcc significantly reduced BPH fecundity under all experimental thermal conditions, indicating to us that *Acc* supports reproduction via fatty acid synthesis. The expansion of BPH biogeography by migration demands efficient energy generation. Again, Acc and Fas produce high-energy fatty acids to support migration. Pesticide-induced increases in BPH body resources has been demonstrated^31^.

Our data also show that JGM treatments led to increased expression of *Hsp 70*, which enhanced thermotolerance and fecundity in thermally stressed, but not in unstressed, BPH. We infer that the influence of Hsp70 on fecundity operates through the general cell protecting mechanisms associated with chaperone proteins and not through a special mechanism unrelated to thermotolerance. The key point here is that we have identified one gene, *Hsp70*, that operates in increasing BPH thermotolerance. Considered in light of the adaptive syndrome, we predict other genes, now awaiting discovery, that confer protection to many cell components also operate in the adaptive syndrome.

Pesticides are powerful driving forces in the evolution of agricultural pests. Our work on genes acting in pesticide-induced thermotolerance and reproduction is a relatively new direction in understanding the evolution of insect relationships with agricultural chemicals. In our view, these relationships can extend far beyond insect resistance mechanisms. Insects have evolved resistance in the field to virtually all major insecticide classes, including the many *Bt* toxins^27^. Agricultural chemicals have been regular features of pest insect habitats for decades. Aside from direct resistance mechanisms, our work is revealing new molecular- and population-level effects of pesticides. We take the immediate significance of this work in the context of the steady, at least for the coming few decades, inevitable increases in global temperatures. Insects continue their evolutionary patterns, adapting to new chemical environments associated with modern agroecology, new thermal environments associated with human-centered global warming and new land use patterns associated with increasing urbanization. The idea that some species have evolved to the point that agrochemicals enhance pest biology presents two major implications to us. One is insects should be viewed as partners in relationships with agricultural chemicals and practices. It follows that insects will continue evolving within these relationships. And second, as these relationships mature, we foresee that more or less large-scale agricultural disasters are not impossible.

## Materials and Methods

### Geographic data

We assessed the rate of BPH expansion in China by collecting geographic range data from 1991 to 2012 from the National Agro-Tech Extension and Service Centre of China (NATESC).

### Mapping and rate of BPH expansion

We used geographic information system (GIS) to map of BPH expansion from 1992 to 2012.

The image analysis was conducted in R using the Portable Network Graphics (png) and raster packages^32^. The png file was read to produce a 3-layer raster object for red-green-blue values, each pixel scaled from 0 to 1. As spatial information was not stored in the png files, the pixel indices were manually inspected to match the two ends of the map’s scale bar. From this, the east-west and north-south distances corresponding to one pixel was determined, yielding the geographical area of each pixel. The R code counted the green pixels and multiplied by the pixel area. Evaluation of errors caused by possible artifacts from the original jpeg compression and issues such as including or excluding the boundary line indicates the method is likely within 3–4% accuracy, based on the independent methods of estimating areas at provincial level and summing.

### Plant, insect and insecticide preparation

Rice (*Oryza sativa* ssp. *japonica*) cv. Yangjing805 seeds were sown in cement tanks (60 × 100 × 100 cm) at the Yangzhou University experimental farm in eastern China (32.3833°N, 119.4167°E). Thirty-day-old seedlings bearing six leaves were transplanted into pots (35 cm diameter, 50 cm tall) and each covered with a cage (insect-proof nylon, 60-mesh (Xinqiao Gauze Co. Ltd., Zhejiang, China). To mimic the typical planting pattern, pots were set up with four ‘hills’, each with four seedlings. Plants were grown to the tilling stage under natural conditions.

Experimental BPHs were obtained from a laboratory population maintained since 2001 at the China National Rice Institute (Hangzhou, China; 32.9000°N, 115.8167°E). The experimental population was maintained on rice cultivar Yangjing805 in an insectary covered with cages under natural conditions in cement tanks (60 × 60 × 100 cm) from April to October; they were maintained in a greenhouse the rest of the year. Prior to the experiments, two generations of BPHs were maintained on Yangjing805 in an insectary with controlled temperature and photoperiod (28 ± 2 °C and L:D 16:8). The agricultural antibiotic, technical JGM (61.7% a.i.), was obtained from Qianjiang Biochemical Co., Ltd. (Haining, Zhejiang, China).

### Experiments

Three hundred third-instar BPH nymphs were released into each experimental pot with 80 mesh screen cages. At 24 h after the insects were placed in the pots, the plants were sprayed with JGM (200 ppm amended with 10% of the emulsifier Tween 20 diluted in tap water, because JGM is water-soluble) (diluting based on total active component of JGM because its active component includes A, B, E, and F) using a Jacto sprayer (Maquinas Agricolas Jacto S.A., Brazil) equipped with a cone nozzle (1-mmdiameter orifice, 45 psi, and flow rate 300 ml/min). Control plants (same developmental stage) were sprayed with a mixture of tap water and the emulsifier. Each treatment was replicated three times (3 pots) and plants were laid out in a randomized pattern. Insects were collected at 48 h after spraying and placed separately in glass jars (diameter 10 cm, height 12 cm) with untreated rice plants to prepare for the RNAi experiments.

### RNA interference

Previous studies demonstrated that *Hsp70* contributes to tzp-induced BPH thermotolerance^15^ and that *Acc* contributes to JGM-enhanced reproduction^20^. We selected these two genes for functional analysis of JGM-induced increases in thermotolerance and reproduction. Total RNA was isolated from 5 adult BPH females using a SV Total Isolation System Kit (Promega Corporation, Madison, WI, USA). Synthesis of first-strand cDNAs was carried out according to the PrimeScriptRT Reagent Kit (TaKaRa Biotechnology, Dalian). The reaction was conducted in a 10 μL total volume containing 0.5 μg total RNA, 0.5 μL PrimeScriptRT Enzyme mix I, 0.5 μL Oligo dT Primer (50 μM), 2 μL random hexamers (100 μM), 2 μL 5X PrimeScript Buffer (for real time-PCR) and RNase–free dH_2_O. The reaction conditions were 37 °C for 15 min, 85 °C for 5 s and 4 °C for 5 min. We synthesized dsHsp70 and dsAcc constructs using a 479 bp fragment (*NlHsp70*) anda 479 bp fragment (*NlAcc*) amplified by PCR using *NlHsp70* and *NlAcc* cDNAs with primers containing the T7 primer sequence at the 5′ end (all primers used in this report are listed in Table 2). The amplification reactions were programmed for 35 cycles at 95 °C for 1 min, 55 °C or 56 °C for 40 s, 72 °C for 1 min, with a final extension step of 72 °C for 10 min. The identities of PCR products were verified by DNA sequencing. Only those sequences in which products from the forward and reverse sequences aligned well (98–99%) were used as templates for dsRNA synthesis. The green fluorescent protein gene (*Gfp*) (ACY56286) was used as a control for the non-specific influence of a dsRNA construct. The dsRNAs were prepared using the T_7_ RiboMax Express RNAi System (Progema, Madison, WI, USA). Sense and antisense dsRNAs generated in separate 20 μl total reaction volumes were annealed by mixing both transcription reactions and incubating at 70 °C for 10 min then cooling to room temperature over a 20 min period. A 2 μl RNase A solution (1:200) and 2 μl RNase-free DNase were added to the reaction mixtures of both transcription reactions and incubated in 37 °C water for 30 min. The dsRNA was precipitated by adding 110 μl 95% ethanol and 4.4 μl 3 M sodium acetate (pH5.2), washed with 0.5 ml 70% ethanol and then dried at room temperature before dissolving in 50 μL nuclease-free water. The purified dsRNAs were quantified by UV at 260 nm and examined by agarose gel electrophoresis to ensure their integrity. Insects were released onto potted rice plants and treated with JGM as just described. Experimental and control 5^th^ instar nymphs (20/treatment) were collected 3 days after JGM treatments. They were treated with RNAi according to Dong *et al*.^33^. In brief, nymphs were transferred to an artificial diet (Fu *et al*.)^34^. Glass cylinders (15 × 2.5 cm) were used as feeding chambers. The liquid artificial diet (40 μL), supplemented with dsRNA, was held between two layers of Parafilm M stretched over the both open ends of the feeding chambers. The cylinders were placed in a biological culture box (under standard conditions) and the middle portion of the cylinders covered with a black cotton cloth to encourage the positively-phototaxic nymphs to the ends of the cylinders where they could penetrate the inner Parafilm layer to access the dsRNA-laced artificial diet. The diet solution was changed every 2 days. A predetermined dsRNA concentration of 0.0625 μg/μl for *Hsp70* and *Acc* (dsRNA at this concentration decreased target gene expression by 70% compared to controls) was used in this experiment. After four days of feeding on the artificial diet with the dsRNA, 60 newly emerged adult females were transferred to biological culture boxes at a high temperature (40 ± 1 °C, standard conditions), and mortality was recorded every 4 h. The experiment was replicated six times (10 females per replicate).

### Thermotolerance and enhancement of reproduction induced by JGM

Experimental and control females were placed in biological culture boxes at a high temperature (40 ± 1 °C) with a 16:8 L:D photoperiod and 75% relative humidity. The suitable temperature for BPH is 25–28 °C^35^. Each treatment and the control was replicated six times (n = 60 females). The number of dead females was recorded at 4, 8, 12, 24, 36, 48, 60 and 72 h exposure to high temperature. The LT_50_ was calculated based on the Gompertz model^36^:


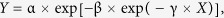


where *Y* is number of dead females, *X* is time (h), α is the upper asymptote, and β and γ are the parameters associated with the mortality rate.

Experimental and control females were placed in biological culture boxes at 34 ± 1 °C and 26 ± 1 °C (because 40 °C is too high for fecundity and unsuitable egg-laying experiment, but can be used in the theromolerance experiment). Thus, we designed three temperature groups for theromolerance and only two groups for fecundity), 16:8 L:D and 70–75% relative humidity. A pair from the same replicate was placed in a glass cup containing untreated rice stems for egg-laying. Each treatment and control was replicated 18 times (18 pairs from the same dsRNA group following the same replicates of treated potted rice). Rice stems were replaced every 2 days. The number of eggs laid on each rice stem was counted under a microscope.

### qRT-PCR analysis

Total RNA from the experimental and control BPHs were extracted and reverse transcribed. Portions (2 μl) of the first-strand cDNA were amplified by qPCR in 20 μl reaction mixtures using a 7500 real-time PCR system (Bio-Rad Co., California, USA), programmed at 94 °C for 2 min, followed by 40 cycles of 94 °C for 5 s, 59.6 °C (*Hsp70)* or 49.4 °C (*Acc*) for 30 s, and 72 °C for 30 s. The BPH Actin-1 gene was used as a reference gene. After amplification, a melting curve analysis was performed in triplicate and the results were averaged. The values were calculated using three independent biological samples. The 2^−ΔΔCT^ method^37^ used for the analysis of relative *Hsp70* and *Acc* expression. Each treatment was replicated three times (five females per replicate).

### Statistical analysis

Normal distributions and homogeneity of variances (determined using the Bartlett test) were verified before performing analysis of variance (ANOVA) tests. A one-way ANOVA was performed to analyze LT_50_ values and reproduction of BPH in the thermotolerance and reproduction study. All data are expressed as mean ± SE. Multiple comparisons of the means were conducted based on the Fisher-protected least significant difference (PLSD). All of the analyses were conducted using the data processing system developed by Tang & Feng (2002)^38^.

## Additional Information

**How to cite this article**: You, L.-L. *et al*. Driving Pest Insect Populations: Agricultural Chemicals Lead to an Adaptive Syndrome in *Nilaparvata Lugens* Stål (Hemiptera: Delphacidae). *Sci. Rep.* **6**, 37430; doi: 10.1038/srep37430 (2016).

**Publisher's note:** Springer Nature remains neutral with regard to jurisdictional claims in published maps and institutional affiliations.

## Figures and Tables

**Table 1:** *F*-statistics for the indicated experiments.

Experiment	*F*-Statistic
LT_50_ values of thremotolerance	*F* = 13.1, df = 4, 25, *P* = 0.0001
Expression level of *Hsp 70* at 40 °C	*F* = 102.1, df = 3, 8, *P* = 0.001
Expression level of *Acc* at 40 °C	*F* = 124.9, df = 3, 8, *P* = 0.0001
The number of eggs laid (NEL) at 26 °C	*F* = 30.7, df = 4, 85, *P* = 0.0001
NEL at 34 °C	*F* = 47.7, df = 4, 85, *P* = 0.0001
Preoviposition perid (POP) at 26 °C	*F* = 20.8, df = 4, 85, *P* = 0.0001
POP at 34 °C	*F* = 8.0, df = 4, 85, *P* = 0.0001
Oviposition perid (OPP) at 26 °C	*F* = 5.9, df = 4, 85, *P* = 0.0003
OPP at 34 °C	*F* = 7.0, df = 4, 85, *P* = 0.0001
Longevity (LGV) at 26 °C	F = 1.1, df = 4, 85, P = 0.34
LGV at 34 °C	F = 1.7, df = 4, 85, P = 0.17
Expression level of *Hsp 70* at 26 °C for JGM + ds*Hsp70*	*F* = 64.4, df = 3, 8, *P* = 0.0001
Expression level of *Hsp 70* at 34 °C for JGM + ds*Hsp 70*	*F* = 155.7, df = 3, 8, *P* = 0.0001
Expression level of *Acc* at 26 °C for JGM + ds*Acc*	*F* = 223.5, df = 3, 8, *P* = 0.0001
Expression level of *Acc* at 34 °C for JGM + ds*Acc*	*F* = 56.8, df = 3, 8, *P* = 0.0001

**Table 2:** Primers used in this research.

Primer	Primer sequence
For quantitative real-time PCR	
Q*NlHsp70*-F	5′-CGATGAGGGCTCTCTATTTG-3′
Q*NlHsp70*-R	5′-CTCGTGGGTTGGACTTGAT-3′
Q*NlAcc*-F	5′-TTACTGATGGCTTGGCTAC-3′
Q*NlAcc*-R	5′-CGACATTACGACCCTGAC-3′
For *Hsp70* and *ACC* dsRNA synthesis	
*NlHsp70*-F	5′-CTGACCATCGAGGATGGCATCT-3
*N*l*Hsp70*-R	5′-ACCGCCTCGTCGGGATTGAT-3′
*NlAcc*-F	5′-TTACTGATGGCTTGGCTAC-3′
*NlAcc*-R	5′-CGACATTACGACCCTGAC-3′
For *Gfp* dsRNA synthesis	
*Gfp*-F	5′AAGGGCGAGGAGCTGTTCACCG-3′
*Gfp*-R	5′-CAGCAGGACCATGTGATCGCGC-3′
